# Early postoperative complications following periacetabular osteotomy: a single-center cohort study on 1,356 consecutive procedures

**DOI:** 10.2340/17453674.2025.44402

**Published:** 2025-08-07

**Authors:** Anne R KRISTIANSEN, Ole OVESEN, Martin H HAUBRO, Anders HOLSGAARD-LARSEN, Søren OVERGAARD, Martin LINDBERG-LARSEN

**Affiliations:** 1 Orthopaedic Research Unit, Department of Clinical Research, University of Southern Denmark; 2 Department of Orthopaedic Surgery and Traumatology, Odense University Hospital; 3 Department of Orthopaedic Surgery and Traumatology, Copenhagen University Hospital Bispebjerg; 4 Department of Clinical Medicine, Faculty of Health and Medical Sciences University of Copenhagen, Denmark

## Abstract

**Background and purpose:**

Periacetabular osteotomy (PAO) is a major surgical procedure, yet data on early postoperative complications and hospitalizations remains limited. We aimed to report postoperative complications within 90 days using the modified Clavien-Dindo classification system. Our secondary aim was to report the peri- and postoperative complications observed in patients with length of hospital stay (LOS) exceeding 4 days or readmitted within 90 days following PAO.

**Methods:**

We identified patients who underwent PAO at a single institution between 2006 and 2021. Patient characteristics, LOS, in-hospital complications, and readmissions within 90 days postoperatively were obtained from our institutional database, patient files, and the Danish National Patient Registry to ensure complete follow up. Minor complications were defined as Clavien-Dindo grades 1 and 2, while major complications were defined as grades 3 and 4.

**Results:**

1,356 consecutive PAO procedures were performed in 1,096 patients with a mean age of 29.3 years (SD 11.1) and 77% females. Minor complications occurred in 499 hips (37%, 95% confidence interval [CI] 35–39) within 90 days of PAO. Only 16 (1.2%, CI 0.6–1.8) major complications were observed. LOS exceeded 4 days in 244 cases (18%) most frequently linked with nausea and emesis in 40 (2.9%). The 90-day readmission rate was 4.4% (CI 2.6–6.2), most commonly linked with pain in 11 (1.0%) and wound infection requiring antibiotics also in 11 (1.0%).

**Conclusion:**

PAO was associated with 37% minor complications within 90 days, while major complications were rare, occurring in only 1.2% of cases. After 18% of PAO procedures, LOS exceeded 4 days, and the 90-day readmission rate was 4.4%.

The periacetabular osteotomy (PAO) was first described in 1988 by Ganz et al. [1] and intended for treating acetabular dysplasia. Over the years, PAO has become a reliable and effective procedure for hip preservation, with extended applicability to other hip disorders, including acetabular retroversion, congenital dislocation of the hip, and Calvé–Legg–Perthes disease [2]. PAO is performed to reduce hip pain, and improve function and quality of life [3,4].

As an invasive and complex procedure involving substantial surgical trauma with a significant surgical stress response, PAO may be associated with complications [1,5,6]. Given these factors, understanding and ensuring patient safety during and after the procedure is crucial. Previous studies on patients undergoing PAO [7,8] reported low-grade complication rates of 32% and 34%, respectively, and high-grade complication rates of 12% and 9%, according to the modified Clavien-Dindo grading system [7,8].

In addition to post-surgical complications, the length of hospital stay (LOS) and readmissions after PAO have significant implications for healthcare providers and patients. Understanding the factors that influence the duration of hospitalization can aid in patient counseling, resource allocation, and overall healthcare planning. Few have studied postoperative early complications and hospitalizations following PAO, which is why there is a need for more knowledge on the frequency of complications, reasons for excessive LOS, and readmission.

The primary aim of our study was to report the overall complications within 90 days after PAO using the modified Clavien-Dindo classification system. Secondarily, we aimed to evaluate complications associated with postoperative LOS exceeding 4 days and readmissions within 90 days after PAO.

## Methods

The guidelines outlined in the “Strengthening the Reporting of Observational Studies in Epidemiology” (STROBE) Statement were followed for reporting this observational retrospective cohort study [9].

### Study design and population

**Figure 1:**
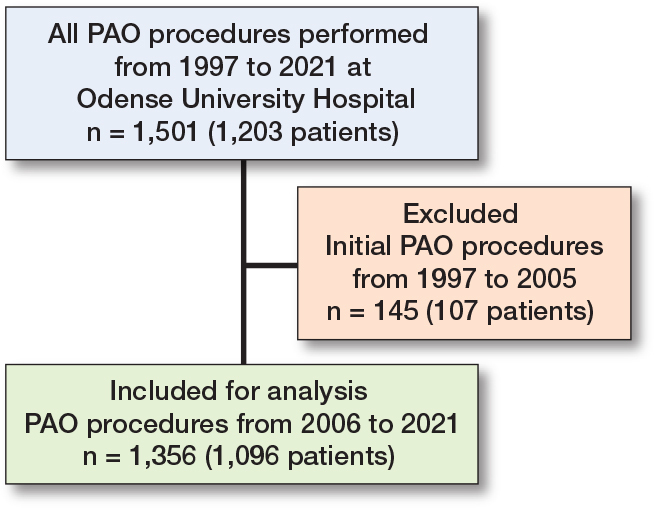
Flowchart of the study population. PAO = periacetabular osteotomy.

This is a retrospective study on patients undergoing primary PAO from 2006 to 2021 at Odense University Hospital (OUH), Denmark. Procedures performed between 1997 and 2005 were excluded to reduce heterogeneity related to evolving surgical techniques, perioperative care protocols, and postoperative recovery pathways during the early phase of PAO implementation (Figure 1). Patients were identified from our institutional PAO database, which has also been used in a previously published study that reported the hip survival outcomes for our entire cohort of PAO procedures [2]. Patients were only included if the triradiate cartilage was closed, based on preoperative imaging.

### Surgical procedure and follow-up

All surgeries were performed by 3 experienced surgeons (SO, OO, MBØ) using a uniform technique via a modified Smith-Petersen approach [10]. No wound drains or local infiltration anesthesia were used. The procedure begins with a skin incision at the anterior superior iliac spine, extending distally 7–10 cm. After fascial incision, the sartorius and psoas muscles are retracted, and the periosteum is elevated medially along the ilium to just below the linea terminalis and further to expose the pubic bone. The ischium is exposed from the anterior and medial side. Osteotomies are performed with the muscles retracted, and after the acetabular fragment has been reoriented it is fixed with 2 screws. Patients were mobilized with crutches on the day of surgery partly weightbearing, allowing 40 kg of weight on the osteotomy side for the first 6–8 weeks. Postoperative radiographs were obtained on the first postoperative day. Follow-up, including clinical assessment and radiographs, was scheduled at 6–8 weeks postoperatively. A 90-day cutoff was chosen to define early complications, consistent with international standards for early postoperative reporting.

### Institutional database and the Danish National Patient Registry

Data on all patients undergoing PAO included: sex, height, weight, BMI, age at surgery, right or left hip, type of underlying hip condition leading to PAO, and degree of OA before surgery. Data was prospectively entered by the surgeon before surgery on every patient. In the case of missing preoperative data in the database, patient records were reviewed. Furthermore, all patient records were scrutinized for data on LOS and readmissions within 90 days.

A retrospective review of patient files could only detect readmissions within the Region of Southern Denmark, which is why we completed data on all readmissions, through additional information from the Danish National Patient Registry (DNPR), Statistics Denmark. The DNPR is a national registry containing all patient admissions, including procedure and diagnosis codes, across all Danish hospitals [11].

### Outcomes

*Primary outcome.* This was the overall complication rate within 90 days post-PAO, classified according to the modified Clavien-Dindo grading system [12] (see Supplementary data).

For the modified Clavien-Dindo grading system: Grade 1 complications did not alter the treatment course and included minor issues such as heterotopic ossification (HO), fever, nausea, constipation, urinary tract infections, wound problems not requiring intervention, pain, and mild anemia not requiring transfusion (hemoglobin [Hb] of <8 mmol/L [male] and <7.45 mmol/L [female]). Grade 2 complications required minor adjustments to the treatment plan, such as the need for antibiotics or additional follow-up for issues like superficial wound problems, transient nerve injuries, delayed union, or anemia necessitating transfusion. Grade 3 complications were more severe, often requiring surgical intervention, and included cases of nonunion, fractures, deep infections, HO requiring surgery, and deep vein thrombosis (DVT). Grade 4 complications were life-threatening or resulted in permanent damage, including osteonecrosis, permanent nerve injury, vascular damage, and pulmonary embolism. Grade 5 complications result in death and were not seen in this study [12]. The Clavien-Dindo classification was applied to each surgical intervention. Complications were registered during the primary hospitalization, at the first follow-up, upon patient contact, or in the case of readmission. In cases where multiple complications were present after the intervention, the highest grade was recorded [13].

*Secondary outcomes.* These were complications registered in patients with LOS > 4 days and patients with readmissions within 90 days. Prolonged LOS was defined by the upper quartile of LOS, which in our study is 4 days, thus we used LOS > 4 as a cut-off for identifying in-hospital complications. Data is presented as follows: (i) all complications occurring within 90 days, (ii) complications registered in patients with prolonged LOS > 4 days, and (iii) complications registered in patients with re-admissions within 90 days (limited to only the worst complication per hip).

### Statistics

Categorical data is reported as numbers with corresponding percentages. Normally distributed data is shown as means with standard deviations (SD), while non-normally distributed data is presented as medians with interquartile ranges (IQR). 95% confidence intervals (CIs) were calculated for all proportions and risk estimates using the Wald method. If patients underwent surgery on both hips each hip was included and analyzed as a separate observation, as all contralateral surgeries were performed at least 6 months apart and typically several years apart, each representing a distinct surgical event [14].

All statistical analyses were conducted using STATA/IC, version 17 (StataCorp LLC, College Station, TX, USA).

### Ethics, funding, data sharing plan, and disclosures

Data processing and storage were registered in the Region of Southern Denmark’s record of data-processing activities. Access to data was requested following the Danish Health Act and was granted by the Region of Southern Denmark, allowing access to patient files without informed consent (case nos. 21/47010 and 21/42892). All data was processed and stored in compliance with the EU General Data Protection Regulation (GDPR) and the Danish Data Protection Act. Due to Danish data protection laws, the data from this study cannot be shared publicly. Access is restricted to authorized personnel and governed by strict privacy regulations to protect patient confidentiality. This study received funding from the Local Tissue Bank (Knoglebanken) to cover expenses for data extraction from the DNPR. The authors have no conflicts of interest to declare. Complete disclosure of interest forms according to ICMJE are available on the article page, doi: 10.2340/17453674.2025.44402

## Results

### Demographics

Our cohort comprised 1,356 consecutive PAO procedures performed in 1,096 patients from 2006 to 2021. 781 (58%) were diagnosed with acetabular dysplasia, 489 (36%) with acetabular retroversion, 41 (3.0%) with congenital dislocation of the hip, and 45 (3.3%) with Calvé–Legg–Perthes disease. The mean patient age at the time of operation was 29.3 years (SD 11.1) and 77% were female (Table 1).

**Table 1:** Baseline characteristics. Values are count and proportion (%) unless otherwise specified

Item	Overall
Number of hips	1,356
Age at operation	
mean (SD)	29.3 (11.1)
range	11–61
Sex	
female	1,042 (77)
male	314 (23)
Body mass index	
mean (SD)	24.5 (4.0)
range	15.4–42.9
missing	3 (0.2)
ASA score	
1	1,035 (78)
2	286 (22)
3	6 (0.5)
missing	29 (2.1)
Side of operation	
right	754 (56)
left	602 (44)
Tönnis arthritis grade	
0	1,304 (96)
1	49 (3.6)
2	2 (0.2)
3	1 (0.1)

### Primary outcome

37% (CI 35–39; n = 499) had minor (grade 1 or 2) complications, 1.2% (CI 0.6–1.8; n = 16) had major (grade 3 or 4) complications, and no deaths occurred (grade 5) within 90 days. 62% (n = 857) of patients experienced no complications (Table 2).

**Table 2:** Overall complications by the modified Clavien-Dindo classification. Only the highest-grade complication per patient

Complication grade	n (%)
No complication	841 (62)
Grade 1	434 (32)
Grade 2	65 (4.8)
Grade 3	13 (1.0)
Grade 4	3 (0.2)
All with complications	515 (38)

### Secondary outcome

**Figure 2:**
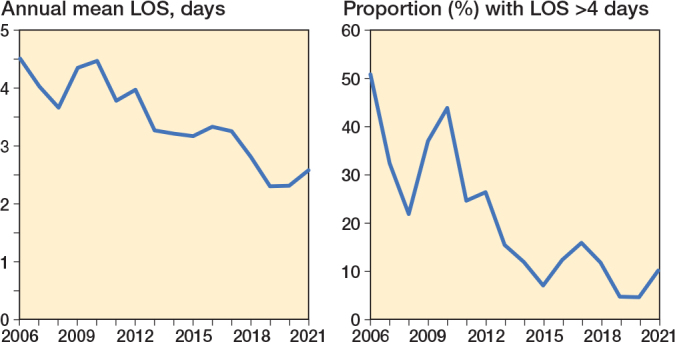
Time-trends in mean length of hospital stay (LOS) and LOS > 4 days during the study period from 2006 to 2021.

The mean LOS was 3.3 days (SD 1.4) decreasing over time to 2.6 days (SD 1.7) by 2021 (Figure 2). LOS > 4 days were observed in 243 (18%) of the 1,356 PAO procedures, whereas 59 (4.4%) were readmitted within 90 days.

### Complications in patients with LOS over 4 days

297 complications were recorded during the primary hospitalizations. Among the 243 cases with a LOS > 4 days, 159 cases (65%) experienced 1 or multiple complications during their hospital stay, while 85 cases (35%) had no recorded complications, despite the extended LOS. In 97 (7.2%) out of all 1,356 PAO procedures, more than 1 complication was observed during the extended LOS; in these cases, only the most severe complication was listed (Table 3). The most frequently observed complication was nausea and emesis, affecting 40 cases (17%) out of 244 with LOS > 4 days. Additionally, 28 cases (12%) experienced pain of such severity that it necessitated the administration of extra analgesics during their hospitalization beyond the planned standard pain management plan.

**Table 3:** Registered complications after PAO procedures with LOS > 4 days. The first column includes all registered complications (multiple per hip). The second column includes only 1 (the worst) complication per hip

Complication	All compli-cations, n (n = 297)	1 registration per hip, n (%) (n = 243)	Comment (examples of complications)
No recorded complication	85	85 (35)	
Nausea/emesis	69	40 (17)	
Pain	73	28 (12)	
Urogenital	21	17 (7.0)	Urinary tract infection, urinary retention
Anemia **^a^** (no transfusion)	15	13 (5.3)	
Meralgia paresthetica	29	10 (4.1)	
Anemia **^a^** (transfusion)	9	9 (3.7)	
Dizziness	22	7 (2.9)	
Gastrointestinal	10	7 (2.9)	Gastritis, abdominal pain
Problems with mobilization	24	4 (1.6)	
Displacement of osteotomy/fracture	3	3 (1.2)	Displacement/fracture, re-operation
Wound oozing	2	1 (0.4)	
Cardiac	2	1 (0.4)	Chest pain
Other	18	18 (7.4)	Skin itching/rash, cramps, fever, anxiety, hematoma, desaturation, anaphylactic shock, drop foot, elevated INR (international normalized ratio), hypotension

a Hemoglobin (Hb) of < 8 mmol/L (male) and < 7.45 mmol/L (female).

### Complications in patients readmitted within 90 days

After 59 (4.4%, CI 3.3–5.6) out of the 1,356 PAO procedures, the patient was readmitted within 90 days. 17 of the 59 cases had more than 1 readmission, but only the first was included in the current analysis. Pain (n = 11) and wound infection requiring antibiotics (n = 11) were the most frequently registered complications observed in the readmitted patients (Table 4).

**Table 4:** Registered complications in patients readmitted within 90 days postoperatively. 59 patients were readmitted; only the first readmission per patient was included in the analysis

Complication	n	Examples of complications
Pain	11	Contusion of the hip
Infection, requiring antibiotics	11	
Infection, requiring revision	8	
Urogenital	4	Urinary tract infection, ureteral stone
Suspected DVT, ruled out	4	
Pulmonary	3	Upper respiratory tract infection, asthma
Reoperation	3	Re-PAO, surgery for hematoma, acetabular fracture
Gastrointestinal	3	Appendicitis, cholecystitis, toxic hepatitis
Other	12	Hematoma, adverse drug reaction, contusion of head/hand, hyperhidrosis, diabetes, spontaneous abortion, complication to heart valve prosthesis

14 patients experienced a second complication/readmission, and 3 a third within 90-days. Reasons were pneumonia, deep vein thrombosis, abscess, unspecified (5), infection (3), hepatitis, diabetes, pulmonary embolism, ear, nose, and throat issues, elbow problems, and abdominal pain.

## Discussion

The primary aim of our study was to report the overall complications within 90 days after PAO using the modified Clavien-Dindo classification system. Secondarily, we aimed to evaluate complications associated with postoperative LOS > 4 days and readmissions within 90 days after PAO.

We found that PAO was associated with only 1.2% major complications (Clavien-Dindo grade 3/4) within 90 days postoperatively. No deaths were reported. However, minor complications (grade 1/2) were observed more frequently (37%). Prolonged LOS > 4 days occurred after 18% of the PAO procedures and the most common complications observed in these cases were nausea and emesis, pain, and urogenital problems. The 90-day readmission rate was 4.4%, with the most common registered complications being pain and wound infection requiring antibiotics.

### Overall complications

A systematic review by Tønning et al. [15], which included 4,260 PAOs, reported major adverse events in 4.3% of patients, and minor adverse events in 14%. Their major adverse event rate was higher than ours at 1.2%, while the minor event rate was significantly lower than ours at 37%. Only 12 of the 29 studies in the review [15] utilized a classification system for complications, with 10 employing the Clavien-Dindo system. This inconsistency in classification methods and smaller sample sizes in the included studies likely contributed to the variability in reported complication rates. The most comparable study to ours was a prospective study of 205 PAO procedures from Zaltz et al. [16], also using the Clavien-Dindo system. They reported complications within 10 weeks comparable to our 90-day follow-up and found 11% low-grade and 3.4% high-grade complications. Their low-grade rate was much lower than ours (37%), potentially due to differences in reporting criteria, as Zaltz et al. did not include pain as a complication, while their high-grade rate was slightly higher than ours (1.2%).

Tan et al. [7] reviewed 7 studies (n = 20–67, 225 PAO procedures) on acetabular retroversion, finding complication rates of 32% for low-grade and 12% for high-grade complications using the Clavien-Dindo system. Common low-grade complications were transient neuralgia, HO, and the need for subsequent surgical procedures. In our study, the complication rates, including HO, delayed union, and non-union, were lower than in other reports [8,9,16], likely due to the short 90-day follow-up period, as delayed union and non-union are often assessed after 6 months. These findings are consistent with those of Lazar et al. [17], who reported delayed healing and non-union rates of 2.3% and 5.6%, respectively, in a subset of the same patient cohort included in the current study (n = 621 hips) with a minimum follow-up of 6 months (mean 1.1 years).

### LOS and readmissions

No other studies have reported complications associated with prolonged LOS. Notably, 1 study by Steinthorsdottir et al. [6] reported a readmission rate of 6.3% within 30 days following PAO, which is somewhat higher than our 30-day readmission rate of 2.6%. Their study, however, was conducted on a much smaller study population (n = 64) than ours.

We found that the LOS decreased over the study period, which is encouraging and may indicate a continuing perioperative learning curve alongside the introduction of fast-track principles [18].

Our data indicates that the primary issues contributing to an extended LOS were related to logistical challenges, as there appears to be no specific medical reason for stays > 4 days. Additionally, symptoms such as pain, nausea, and emesis are frequently reported in patients during hospitalizations > 4 days. Hence, better pain management and management of opioid-related side effects are key factors for future improvement of the perioperative pathways of PAO patients.

### Strengths

The main strength of this study is its large cohort (1,356 PAO procedures) compared with previous studies examining risks of early postoperative complications after PAO [15]. Furthermore, this study is the first to report LOS and readmission risk, which is of importance for patients and preoperative planning. Another strength of this study is that it does not rely on procedure and diagnostic codes alone in the index population but uses prospectively collected data and reviews of patient records for specific data. This ensures the identification of specific complications postoperatively. The follow-up is enhanced by supplementary data from the DNPR [11], which is a high-quality nationwide register, on all readmissions in Denmark. The DNPR has previously been validated and shown to be reliable for identifying hospital admissions and surgical procedures in Denmark. However, it is limited to hospitalizations in Denmark and with the risk of occasional misclassifications or lack of detail in diagnostic coding [11].

### Limitations

A limitation is the studies’ retrospective design, which may lead to underestimation of complications depending on the completeness of available documentation. Minor complications are potentially underreported, particularly altered sensation in the lateral femoral cutaneous nerve. This complication is common but mild and rarely documented, as complications were registered only during the primary hospitalization, at the first follow-up, upon earlier patient contact, or in the case of readmission [19].

Another limitation is that we solely focus on complications within 90 days postoperatively, which compromises comparison with some previous studies using longer follow-up periods. However, we consider a 90-day follow-up period to be appropriate for capturing early complications associated with the PAO procedure and the perioperative period.

The 15-year study period may reflect general evolving perioperative practices and improvements in care that could influence LOS. While this period was necessary to ensure an adequate sample size, it introduces potential temporal variability in LOS that should be considered when interpreting the findings.

Major complications, such as revision PAO and subsequent surgeries, are included in our analysis. However, our 90-day follow-up period may result in a lower reported incidence of high-grade complications compared with studies with longer follow-up durations. Given the variability in follow-up periods and the potential for additional complications over time, direct comparisons with these studies [8,16,20,21] should be made with caution. Longer follow-up durations in other studies [8,20,21] might capture more or different complications than those observed in our study. However, we have previously reported our long-term results after PAO addressing the risk of reoperations and conversion to total hip arthroplasty [2]. Hence, the aim of the current study was to focus solely on perioperative complications in the early postoperative phase.

Finally, although all contralateral surgeries were performed at least 6 months apart and typically several years apart, each representing a distinct surgical event, within-subject dependency cannot be entirely ruled out. This may, to some extent, affect outcomes such as length of stay or readmission risk, and should be considered when interpreting the results.

### Conclusion

We found a low rate of major complications (1.2%) and no deaths within 90 days after PAO surgery. However, 18% had a hospitalization of > 4 days and 37% experienced minor-grade complications, primarily nausea and emesis, pain, and urogenital problems. The 90-day readmission rate was only 3.5%.

*In perspective,* although PAO is a safe procedure and most complications are minor, continued efforts to reduce complications through optimization of surgical technique, perioperative care including pain management and opioid-related side effects, and institutional experience should remain a key focus for future clinical practice and research.

### Supplementary data

The Clavien-Dindo grading system is available as supplementary data on the article page, doi: 10.2340/17453674.2025.44402

## Supplementary Material



## Notes

MLL initiated this study. OO and SO performed most of the PAO procedures and recorded data in the database at OUH during the study period. ARK, MLL, OO, AHL, and SO contributed to the initial outline and methodology of the study. ARK and MLL developed the analysis plan and drafted the initial protocol and manuscript. All authors reviewed and approved the final protocol and manuscript. ARK and OO conducted the supplementary data collection. ARK performed the statistical analyses.

Odense Patient Data Explorative Network (OPEN) contributed with methodological advice.

Handling co-editors: Paul Gerdhem and Philippe Wagner

*Acta* thanks Jan Erik Madsen and Sören Toksvig-Larsen for help with peer review of this manuscript.
