# Supplementary material for: Early postoperative complications following periacetabular osteotomy: a single-center cohort study on 1,356 consecutive procedures
Source: Acta Orthop. 2025 Aug 7;96:595–600. doi: 10.2340/17453674.2025.44402 (PMC12337780; doi:10.2340/17453674.2025.44402)
Supplement: Supplementary file 1 [file ActaO-96-44402-s1.pdf]

Supplementary data

Grade	Definition	Specific complications
**1**	A complication that requires no treatment and has no clinical relevance; there is no deviation from routine follow-up during the postoperative period; allowed therapeutic regimens include: antiemetics, antipyretics, analgesics, diuretics, electrolytes, antibiotics, and physiotherapy	Asymptomatic Grade I or II heterotopic ossification; postoperative fever, nausea, constipation, minor UTI; wound problem not requiring a change in postoperative care
**2**	A deviation from the normal postoperative course (including unplanned clinic visits) that requires outpatient treatment: either pharmacologic or close monitoring as an outpatient	Superficial wound infection (additional clinic visits); transient neurapraxia from positioning or surgical retraction that resolves under close observation; nerve palsy requiring bracing and close observation (complete resolution); trochanteric delayed union
**3**	A complication that is treatable but requires surgical, endoscopic, or radiographic interventions or an unplanned hospital admission	Trochanteric nonunion; fracture;deep infection; surgical hematoma; clinically significant heterotopic ossification that requires surgical excision; deep vein thrombosis (admission and anticoagulation)
**4**	A complication that is life threatening, requires ICU admission, or is not treatable with potential for permanent disability; a complication that requires organ resection (THA)	Osteonecrosis; permanent nerve injury; major vascular injury; pulmonary embolism; CNS complications; organ dysfunction
**5**	Death	

The generic Clavin Dindo classification system for reporting complications after orthopedic surgery as validated by Sink et al. [12].
